# Crystal structure of the DNA-binding domain of Myelin-gene Regulatory Factor

**DOI:** 10.1038/s41598-017-03768-9

**Published:** 2017-06-16

**Authors:** Xiangkai Zhen, Bowen Li, Fen Hu, Shufeng Yan, Gabriele Meloni, Huiliang Li, Ning Shi

**Affiliations:** 1 State Key Laboratory of Structural Chemistry, Fujian Institute of Research on the Structure of Matter, Chinese Academy of Sciences, Fuzhou, 350002 China; 2 Department of Chemistry and Biochemistry, University of Texas at Dallas, Richardson, TX 75080 USA; 3 Wolfson Institute for Biomedical Research, University College London, Gower Street, London, WC1E 6BT UK

## Abstract

Myelin-gene Regulatory Factor (MyRF) is one of the master transcription factors controlling myelin formation and development in oligodendrocytes which is crucial for the powerful brain functions. The N-terminal of MyRF, which contains a proline-rich region and a DNA binding domain (DBD), is auto-cleaved from the ER membrane, and then enters the nucleus to participate in transcription regulation of the myelin genes. Here we report the crystal structure of MyRF DBD. It shows an Ig-fold like architecture which consists of two antiparallel β-sheets with 7 main strands, packing against each other, forming a β-sandwich. Compared to its homolog, Ndt80, MyRF has a smaller and less complex DBD lacking the helices and the big loops outside the core. Structural alignment reveals that MyRF DBD possess less interaction sites with DNA than Ndt80 and may bind only at the major groove of DNA. Moreover, the structure reveals a trimeric assembly, agreeing with the previous report that MyRF DBD functions as a trimer. The mutant that we designed based on the structure disturbed trimer formation, but didn’t affect the auto-cleavage reaction. It demonstrates that the activation of self-cleavage reaction of MyRF is independent of the presence of its N-terminal DBD homotrimer. The structure reported here will help to understand the molecular mechanism underlying the important roles of MyRF in myelin formation and development.

## Introduction

Myelin-gene Regulatory Factor (MyRF, also known as MRF, Gm98 or C11Orf9 in human) is considered one of the master transcription factors for myelin production by oligodendrocytes in the central nervous system, which permits action potentials to propagate by saltatory conduction along axons and is very important for powerful brain functions. MyRF is only expressed on the terminal differentiation of oligodendrocytes and controls myelin gene expression directly. When MyRF is deleted, the expression of many myelin related proteins cannot be detected^1^. Loss of myelin results in many severe diseases such as multiple sclerosis^2^, leukodystrophies^3^ and defect in motor learning capability^4^.

MyRF was revealed as a transmembrane (TM) protein located at the endoplasmic reticulum (ER) membrane^5^. Its cytoplasmic portion contains an N-terminal proline-rich region and a following DNA binding domain (DBD) which can be auto-cleaved from the ER membrane by a region between the DNA-binding and TM domains. This region is homologous to bacteriophage tailspike proteins and is named ICA (Intramolecular Chaperone Auto-processing)^6^ or ICD (intramolecular chaperone domain)^7^ which has been found in eukaryotic proteins for the first time. Overexpressed MyRF possess a smaller molecular weight than calculated due to auto-cleavage. It is not clear whether the auto-cleavage mechanism of MyRF is the same as observed for the tailpike protein in the maturation of bacteriophage endosialidases^8^. After cleavage, the N-terminal part of MyRF releases from the ER membrane and enters the cell nucleus guided by the NLS signal within its sequence to activate myelin gene expression. The C-terminal cleavage product of MyRF remains on the ER membrane and its function remains elusive. It is clear that MyRF is a membrane-bound transcription factor (MBTF) cleaved with the novel bacteriophage tailspike-like self-cleavage mechanism^6, 7, 9^.

The DBD of MyRF is the main focus for its transcriptional regulation function. It was predicted to be an Ig-fold transcription factor and is homologous to the yeast sporulation-specific transcription factor Ndt80. However, the size of MyRF DBD is more close to the typical Ig-fold transcription factors while Ndt80 was found to be more complex and showing more extensive contacts with DNA. Thus, the interaction of MyRF DBD with DNA may be different from Ndt80. Moreover, previous reports showed that MyRF DBD functions as a trimer^6^, while Ndt80 binds with DNA as a monomer^10^. Recently bioinformatic studies revealed the preferential DNA binding target of MyRF DBD^7^, which is very different from the Ndt80 target sequence. Thus their DNA binding properties ought to be different too. The details of the interaction between MyRF and its target DNA still need to be investigated. Further studies will also help to understand the molecular mechanism of MyRF co-operating with other transcription factors such as Sox10 and Olig2 during oligodendrocyte development and myelin formation^11, 12^.

In this report, we solved the crystal structure of MyRF DBD and demonstrate that it forms a trimer in the crystal lattice. Unlike Ndt80, MyRF DBD may only interact with the major groove of target DNA. The mutations designed based on our structure disrupted trimer formation but had no effect on auto-cleavage. Our data will help to understand the detailed molecular mechanism of myelin gene transcriptional regulation.

## Results and Discussion

### Overall structure of MyRF DBD

The expressed MyRF fragment (351–717 plus N-terminal his tag and linker) showed a smaller molecular weight than calculated (33 vs. 45 kDa) due to auto-cleavage, which matches with the previous reports^6, 7^. Using limited proteolysis, we generated a proteolytically resistant DBD core that is about 24kD. This DBD core was purified and crystallized.

**Figure 1:**
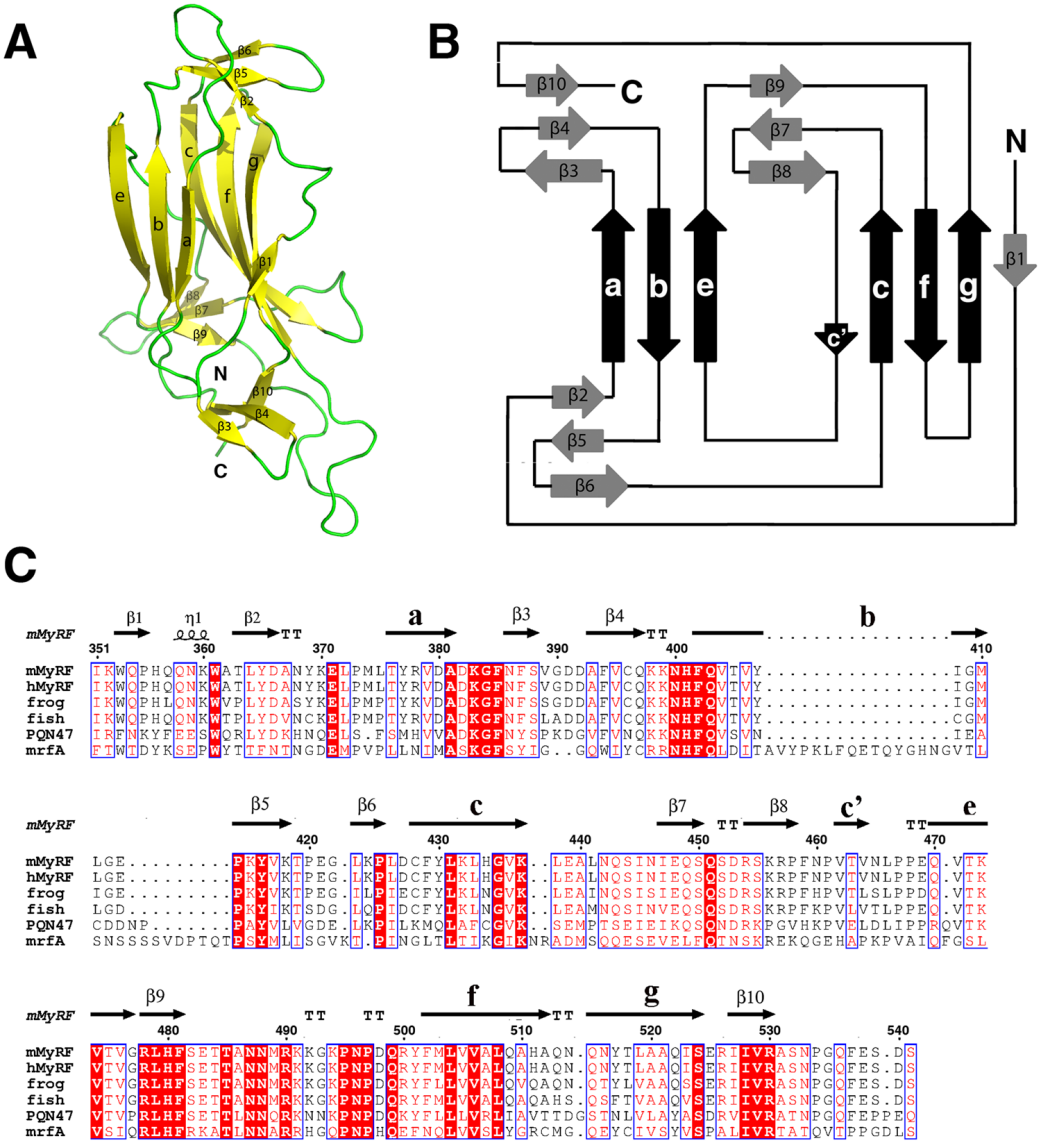
Structure of the MyRF DNA binding domain (DBD). All the strands are labeled according to their occurrence in the primary sequence and the standard nomenclature of Ig- fold^13^. (**A**) Overview of MyRF DBD structure (351–532). The strands and loop are colored yellow and green respectively. (**B**) Topology diagram of MyRF DBD. The conserved s-type Ig-fold strands are indicated in black, while the peripheral outer strands, in gray. (**C**) Secondary structure and sequence alignment of MyRF DBD. Mouse MyRF is aligned with sequences from other *vertebrate* (human, frog and fish), *nematode* (PQN47) and *Dictyostelium* (mrfA). The residue numbering along the top refers to the mouse MyRF.

The structure was determined for the selenomethionyl substituted crystals by using single anomalous diffraction (SAD) phasing from 4 ordered Se atoms per molecule of 210 residues (see Materials and Methods). The resulting experimental electron density map calculated was of sufficient quality to build an initial chain trace and assign the amino acid residues. The structural model has been completed and refined to 2.46 Å resolution (R_work_ = 18% and R_free_ = 23%). The space group is P321 (104.0, 104.0, 46.7, 90, 90, 120), with one molecule per asymmetric unit. Of the 210 amino acids corresponding to the DBD, only residues 351–532 were clearly visible in the electron density map (Fig. 1A).

MyRF DBD is highly conserved in vertebrates (Fig. 1C) and its structure shows a typical Ig-fold architecture. The main part of MyRF DBD consists of two antiparallel β-sheets that have three strands (a, b and e) and five strands (c’, c, f, g and β1) respectively, labeled according to the standard nomenclature of Ig-fold^13^. MyRF DBD can be classified as an s-type Ig-fold, although the 4^th^ switched strand c’ is not obvious. Instead, the N-terminal β1 strand is located next to the g strand of the c–f–g β-sheet, as observed in Ndt80. The sheets pack against each other, forming a β-sandwich with a “Greek key” topology (Fig. 1B). At the end of the β-sandwich, the sheets pack tightly, forming a compact barrel-like structure (Fig. 1A). In addition, there are nine outer strands between the core strands, which are numbered according to their occurrence in the primary sequence. Each group of 3 of these outer strands forms a sheet at the end of core β-barrel. The ribbon representation of the DBD and its topology are illustrated in Fig. 1A and B, respectively.

Generally, Ig-fold proteins do not display high identity to one another at the primary sequence level, although they are similar in their 3D architecture. Consistent with this, MyRF DBD sequence does not show high homology with other members of Ig-fold family, but the structures with similar topology are found in the Protein Structure Database.

### Comparison with other Ig-fold transcription factors

**Figure 2:**
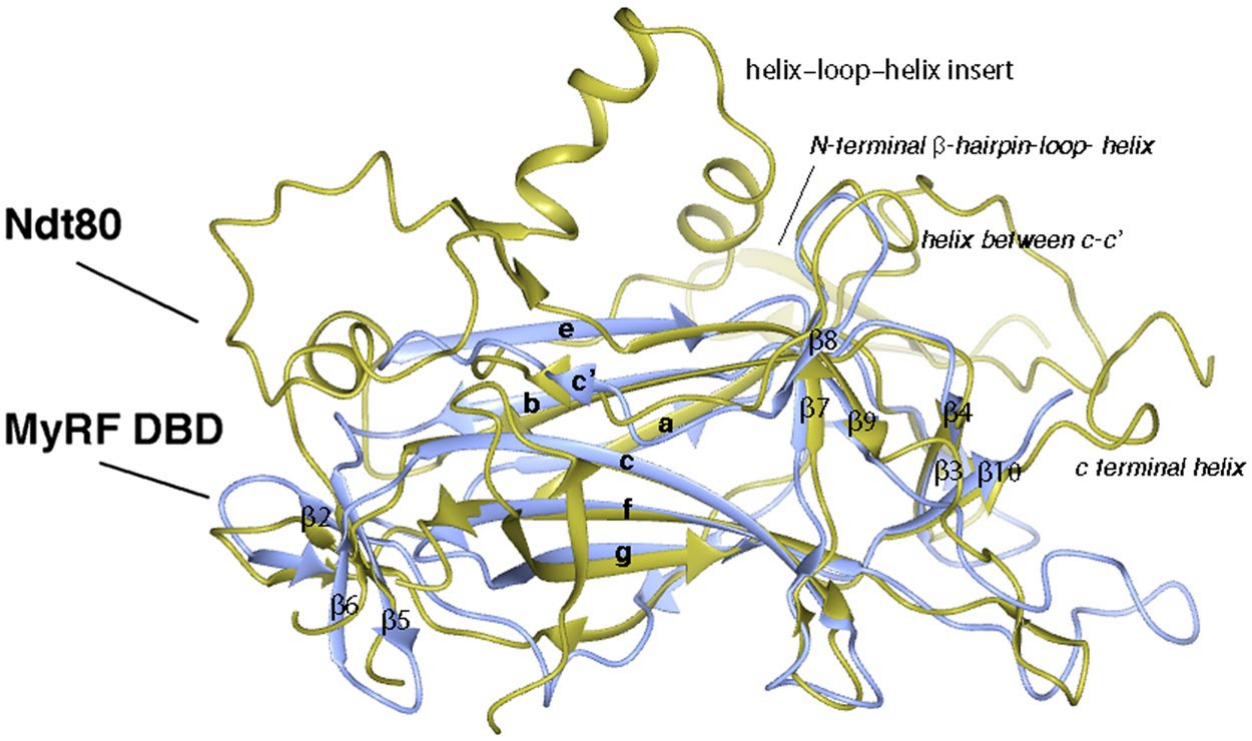
The superimposed structures of MyRF DBD and Ndt80 (PDB code: 1MNN) colored in ice blue and gold respectively. The figure was generated using CCP4gm software.

MyRF DBD is similar in topology to the Ndt80 transcription factors (Fig. 2), as demonstrated by being the best match in a DALI search^14^ with an root mean square deviation (RMSD) of 2.1 Å for 162 C^α^ atoms and 16% sequence identity. Although MyRF DBD and the core of the Ndt80 can be well aligned with each other, Ndt80 is significantly larger than MyRF DBD and other Ig-fold transcription factors. MyRF DBD mainly consists of β-strands. No helical segment and big loop between the core strands are observed, which makes it smaller and less complex than Ndt80. In Ig-fold proteins, the outer strands in the sheets, and the loops between the core strands in particular, are flexible and can be relocated in different proteins without perturbing the core structure of Ig-fold. Without the N-terminal β-hairpin-loop- helix insert, the helix–loop–helix inserts in the c’–e loop, the helix in the C terminal and the helix in the c–c’ loop (which are involved in the direct interaction with target DNA and form an essential sequence-specific recognition site for Ndt80 interacting with target DNA), the interaction of MyRF DBD with target DNA should be different than the one of Ndt80. The fact that MyRF shows less contact with target DNA than Ndt80 indicated that it may bind DNA as a multimer or cooperate with other transcription factors to fulfil its myelin gene activation function.

The second best match of the Ig-like transcription factors in the DALI search was with the members of p53^15, 16^ family, resulting in an RMSD of 2.9 Å for 112 C^α^ atoms and 13% sequence identity. Although the core strands of p53 are shorter than MyRF DBD, 7 core strands of them can be aligned together while the outer strands between the core strands are different. Their DNA binding sites also are different. The other transcription factors resulting from the DALI search showing high degree of structural homology to MyRF DBD include members of STAT^17^, Runx^18^. Some other Ig-fold proteins, such as the Cholesterol-binding Protein^19, 20^, ATP binding part of ABC Transporter^21^ and sugar-binding domain of β-galactosidase^22^, have even higher score in the DALI search than p53, STAT and Runx, but they do not belong to the transcription factor family.

#### Oligomization of MyRF DBD

**Figure 3:**
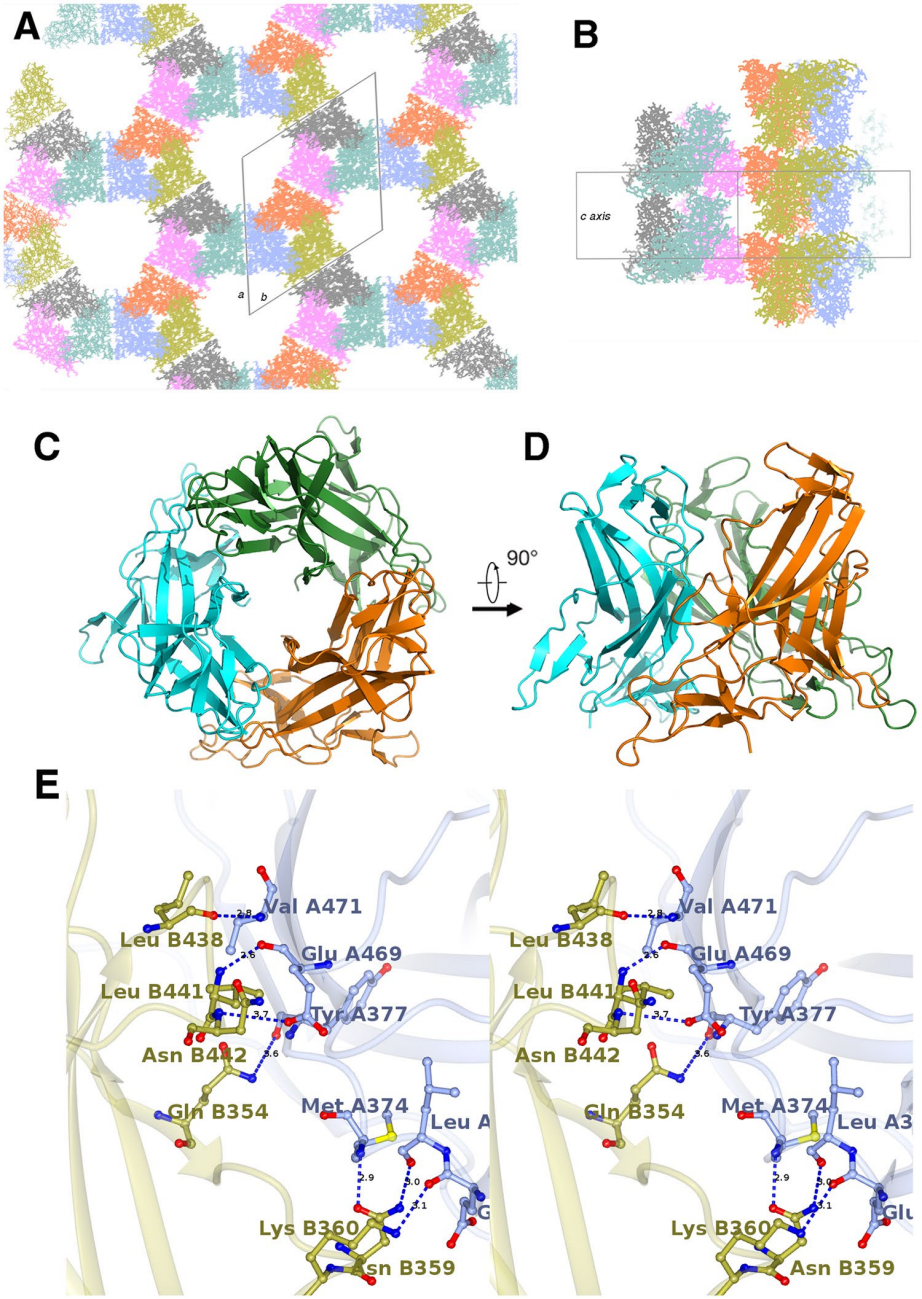
Oligomerization of MyRF DBD in crystal. (**A**) Crystal packing of MyRF DBD viewed from the c axis. Unit cell is shown in black lines. (**B**) Side view of crystal packing. (**C**) Cartoon diagram of MyRF DBD trimer (crystallographic symmetry-related protomers) (**D**) Side view of MyRF DBD trimer. (**E**) Stereo view of detailed trimeric interactions between 2 neighboring protomers. The residues involved in the hydrogen bond interactions are shown with a stick model; nitrogen and oxygen atoms are colored blue and red respectively. 2 neighboring protomers are illustrated and colored ice blue and gold respectively.

MyRF DBD forms a trimer with a crystallographic three-fold symmetry axis in the crystal (Fig. 3A–D). The trimer interface buries a 1497.6 Å^2^ surface area, accounting for 14.5% of total monomer surface area (http://pdbe.org/pisa/). The trimer interaction is mainly mediated by 2 contact regions. Residues 438–442 of the loop between strand **c** and **β7** from one protomer form 3 hydrogen bond interactions with residues 469–471 of strand **e** from the neighboring protomer. Residues 354–360 of the loop between strand **β1** and **β2** from one protomer form 4 hydrogen bond interactions with residues 371–377 of the loop before strand **a** from the other protomer (Fig. 3E). In addition, Arg378 and Lys473 in one of the protomers are positioned in close proximity to E525 and D392 in the other protomer respectively, creating electrostatic attraction that strengthens the trimer formation.

**Figure 4:**
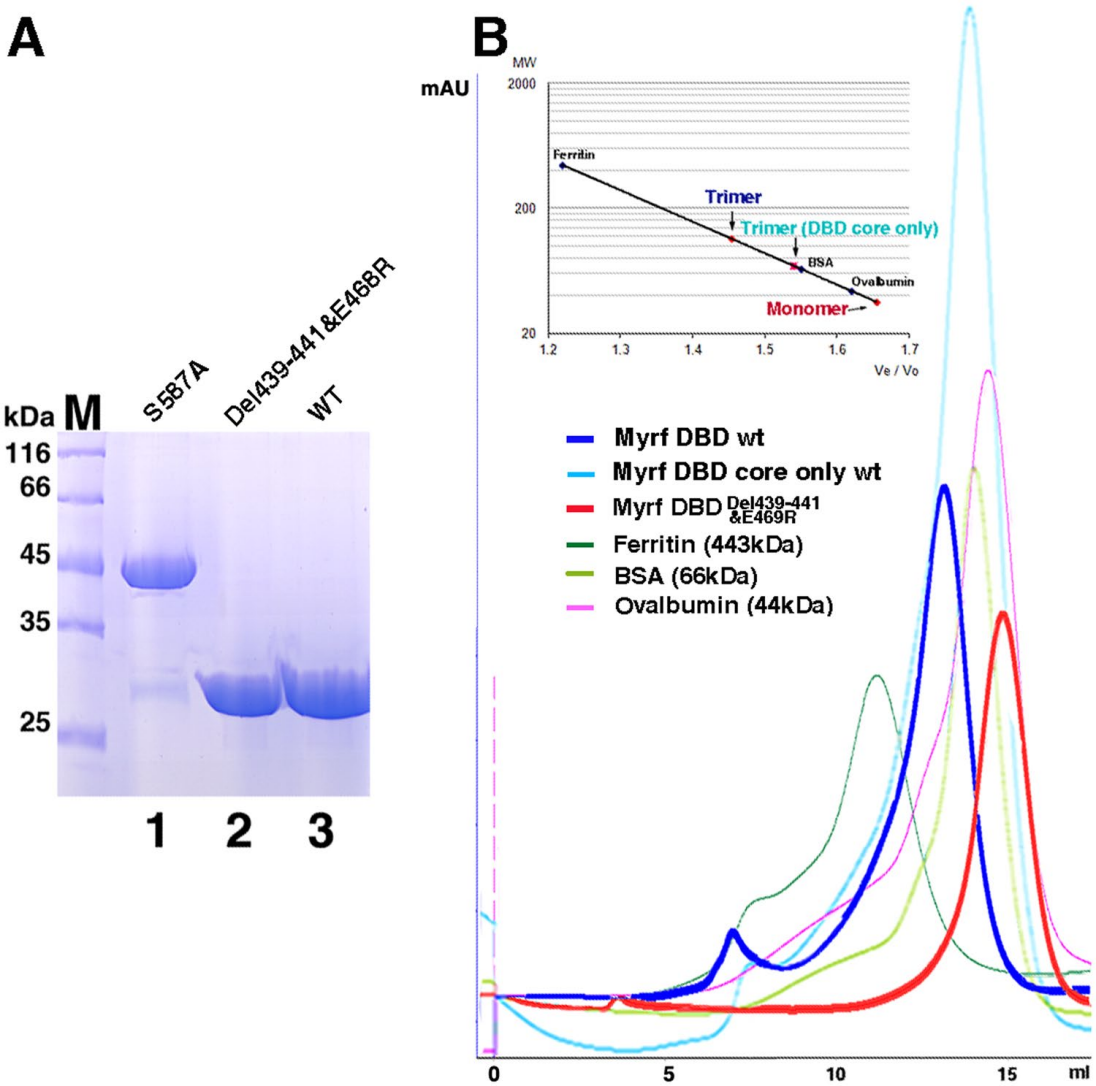
MyRF DBD mutation disturbs trimer formation but does not affect auto-cleavage of MyRF. (**A**) The auto-cleavage of MyRF and its mutants were analyzed by 12% SDS-PAGE. M: protein marker. (**B**) Elution profiles from gel filtration chromatography of MyRF DBD and its mutant. Calibration curve of proteins with known molecular weights (ferritin (443 kDa), bovine serum albumin (BSA; 66 kDa), chicken ovalbumin (44kD); Sigma-Aldrich) was shown. The molecular weight of these proteins was plotted against their calculated Ve/Vo and fitted by exponential regression analysis. On the basis of the calculation, the Ve/Vo of wild type MyRF DBD results in a molecular weight of 110 kDa, and the Ve/Vo of its mutant results in a molecular weight of 35 kDa, indicating the trimeric and the monomeric form of protein respectively. The Ve/Vo of wild type MyRF DBD core only (after treated by limited proteolysis of trypsin) results in a molecular weight of 70 kDa, indicating the trimeric form too.

The PISA program predicts that MyRF DBD forms a metastable trimer. The molecular weight of MyRF DBD after auto-cleavage (351–586 plus N-terminal his tag and linker) is similar to the N-terminal fragment (StrepII-MyRF-319:577) reported^6^. All monomers run at a similar position on SDS-PAGE (Fig. 4A, lane 3). On gel filtration, MyRF DBD eluted as a single peak corresponding to a trimer (Fig. 4B, blue peak). Moreover, even the proteolytically resistant MyRF DBD core only (after treated by limited proteolysis of trypsin) still maintains as a trimer (Fig. 4B, cyan peak).

Previous studies have shown that full-length MyRF forms a trimer before cleavage. The ICA domain was thought to play the main role in MyRF trimer formation due to its C-terminal trimeric helix bundle. It is known that trimerization is required for the auto-cleavage reaction in ICA containing phage tailspike proteins^8^. Our structure indicates the N terminus of MyRF (which contains the proline-rich and DBD region only) after auto-cleavage can still keep the trimeric architecture, in agreement with previous results^6^. As observed in the trimer structure of MyRF DBD, the residues 469–471 and 438–442 lie on the trimer interfaces and form 3 hydrogen bonds between protomers. Based on this structural information, the site-directed mutant MyRF DBD E469R&439-441del was designed. The result of size exclusion chromatography showed that E469R&439-441del mutant disturbed the trimer formation (Fig. 4B, red peak). The crystal structure of bacteriophage K1F endosialidase tailspike protein shows its ICA domain and endosialidases upstream are connected by a triple β-helix. In this triple β-helix, three polypeptide chains wind around a common threefold symmetry axis. Proper assembly of this triple–β-helical fold depends on the trimeric helix bundle on the ICA C-terminal. The cleavage reaction of ICA acts as a control mechanism for generating a correctly folded protein. Only if endosialidases domain upstream of triple β-helix folds to a proper trimer, and the side chains of the key residues involved are in the right position, the self-cleavage reaction can occur^8^. Surprisingly, the mutation E469R&441-443del in MyRF did not affect its self-proteolytic processing, although the mutation disturbed the formation of MyRF DBD trimer (Fig. 4A, lane 2). It demonstrates that the activation of self-cleavage of MyRF is independent of its N terminal DBD trimer. The proper assembly of the triple–β-helical fold of MyRF, which depends on the ICA and its C-terminal trimeric helix bundle, triggers a serine-lysine catalytic dyad to activate the self-proteolysis. Consistent with this, the DBD of MrfA (the ortholog of MyRF in Dictyostelium) is separated from the triple β-helix of ICA domain by a large disordered fragment, resulting in the difficult to affect each other. The mutants in the trimeric helix bundle of ICA C-terminal, which disturb the trimer assembly, prevented the self-cleavage of MyRF^6^. The control mechanism of self-cleavage reaction of MyRF and its relationship with generating a correctly folded trimeric MyRF DBD still need to be investigated.

#### Predicted DNA binding

**Figure 5:**
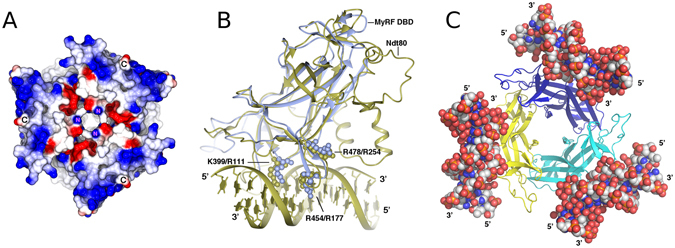
The electrostatic surface of MyRF DBD and the superposition of MyRF DBD with Ndt80 shows potential DNA binding region. (**A**) The electrostatic surface potential of MyRF DBD shows the electropositive region for potential DNA binding. The NH2- (N) and the –COOH (**C**) terminus of MyRF DBD are marked. (**B**) Superposition of MyRF DBD and Ndt80 shows the region that may be involved in the interaction between MyRF DBD and its target DNA. The residues K399, R454, R478 (MyRF) and R111, R177, R254 (Ndt80) are shown as a sphere model. MyRF DBD and Ndt80 colored in ice blue and gold respectively. (**C**) The modeling of MyRF DBD trimer binding with its target DNA. The DNA molecules are shown as a sphere model.

The electrostatic surface potential measurement of MyRF DBD structure reveals a highly electropositive surface owing to the presence of lysine and arginine residues at the bottom of the β-barrel core (Fig. 5A). Superposition of MyRF DBD and Ndt80 showed that these residues may be involved in the interaction of the protein with DNA (Fig. 5B). In particular, 3 conserved alkaline residues in MyRF (K399, R454 and R478) are superimposable with the residues of Ndt80 (R111, R177 and R254) interacting at the major groove of DNA, implying that they may be involved in interaction with DNA too (Fig. 5B). The individual point mutants of these residues dramatically decreased MyRF DNA binding capacity^7, 23^. The aligned structures show that MyRF DBD may only have 4 binding sites, while Ndt80 possess 6 binding sites with the target DNA. The 2 extra binding sites of Ndt80 are located at the minor groove of DNA and come from the extra N terminal β-hairpin-loop-helix and helix–loop–helix inserts in the c’– e loop. All of other 4 binding sites are located at the major groove of DNA. The comparison of their structures indicates that the MyRF DBD monomers have lower binding affinity to DNA than Ndt80. Their differences in binding residues and the position suggest they may recognize different target DNA sequences.

#### Trimer-DNA binding

MrfA, the ortholog of MyRF in Dictyostelium was reported to bind DNA via three distinct elements with 3–5 bp spacing. Mutagenesis studies showed that all three elements are needed for optimal expression *in vivo* ^23^. Previous studies also suggested that MyRF is functional as a trimer^6, 7^, though some of MyRF binding sites identified by ChIP-Seq only had a single binding motif^7^. Consistent with these results, our crystal structure shows MyRF DBD tends to assemble into a homo-trimer. The fact that the promoters of target genes contain different number of the MyRF binding motifs suggests that different genes may be regulated differently. How this trimer interacts with three elements of target DNA is still unclear. According to the model of a single MyRF DBD interacting with DNA, the DNA molecule should bind at the bottom of a trimer. However, it seems difficult to arrange three DNA elements on 3 protomers of a trimer simultaneously with 3–5 bp spacing only (Fig. 5C). Generally, transcription factors interact with DNA as monomers, dimers or tetramers while homo-trimeric transcription factors are rare. Well characterized homo-trimeric transcription factors are heat shock factor 1^24^ and ArgR^25^. Their DBDs do not form trimers directly, but are connected to a trimeric domain by a flexible linker thereby being able to bind on 2 or 3 tandem and adjacent elements at the same time. MyRF is the first transcription factor discovered so far in which the DBD forms a homo-trimer by itself. The relative position of the protomer’s DNA binding sites does not allow major structural changes, otherwise, the MyRF DBD trimer would disassemble. How MyRF activates the expression of its target genes in a trimeric manner is still unclear and further studies are required.

MyRF may cooperate with other transcription factors to control myelin gene expression in oligodendrocytes. It induces a cohort of genes that work together to wrap axon myelin sheathes in the central nervous system. Our structure shows that the N-terminus of MyRF DBD is located at the bottom of the β-barrel core and close to its DNA binding sites (Fig. 5A). Thus the N-terminal proline-rich region, located before the DBD of MyRF, may interact with other transcriptional factors directly. The MyRF homo-trimer can either be stabilized or destabilized by these interactions to regulate the myelin genes expression.

In conclusion, we presented a high resolution structure of MyRF DBD protein with an Ig-fold architecture which consists of two antiparallel β-sheets with 7 main strands, packing against each other, forming a β-sandwich. The structure of MyRF DBD is similar to the core of Ndt80, but with less interaction sites and a different DNA recognition sequence. The structure reveals that MyRF DBD forms a trimer, in agreement with the previous reports demonstrating that MyRF DBD is functional as a trimer. The mutant designed based on the structure demonstrates that the activation of self-cleavage of MyRF is independent of its N terminal DBD trimer. The electrostatic surface potential analysis and comparison with the structure of the complex of Ndt80 and DNA reveal the possible interaction sites between MyRF DBD and DNA, thereby providing molecular mechanistic insight into its transcriptional regulation.

## Methods

### Expression plasmids

Mouse MyRF DNA fragment (residues 351–717, containing DBD and ICA) was amplified from a full length gene clone (NP_001028653.1) and sub-cloned into the modified pRSFDuet-1 vector (Novagen) with an N-terminal 6xHis tag and a flexible linker. Point mutations were introduced using the QuikChange protocol (Stratagene). All constructs were confirmed by DNA Sanger sequencing.

### Protein expression and Purification

The recombinant MyRF was obtained by expression in *Escherichia coli* BL21 (DE3) strain (Novagen) induced with 0.5 mM isopropyl-ß-D-thiogalactopyranoside (IPTG) for 20 hr at 16 °C. The clarified cell lysate after lysis (50 mM Tris-HCl, pH 8.0, and 150 mM NaCl, 1 mM PMSF, Sigma) obtained by sonication, and sequent centrifugation, was incubated with nickel-sepharose affinity resin (GE Healthcare) and washed with lysis buffer. The recombinant protein was analyzed by limited proteolytic mapping, and then subjected to digestion with trypsin (Worthington). At first, the enzyme was dissolved in a buffer of 50 mM Tris-HCl at pH 8.0, and 20 mM CaCl_2_ to make 1 mg/ml trypsin stock solution, and aliquots were frozen at −20 °C. Subsequently the enzyme was added to the purified MyRF protein at a trypsin to protein (w/w) ratio of 1:500,000 in a buffer of 50 mM Tris-HCl at pH 8.0, and 150 mM NaCl. The reaction was incubated at 4 °C for 30 minutes and was terminated by the addition of 10-folds excess of soybean trypsin inhibitor (Sigma) to the reaction mixture. The stable fragments were subsequently purified by size exclusion chromatography on a Superdex-75 column (GE Healthcare) pre-equilibrated in a buffer of 20 mM Tris-HCl at pH 7.4, 150 mM NaCl and 1 mM DTT. Peak fraction was concentrated to 20 mg/ml for crystallization.

Selenomethionine-derivatized MyRF was obtained in *Escherichia coli* BL21 (DE3) strain using methionine pathway inhibition at 289 K. The procedure for protein expression was similar to the native protein except for the cell culture that was performed in M9 medium containing 0.4% glucose, 2 mM magnesium sulfate, 0.1 mM calcium chloride, and 50 µg/l kanamycin. Cells were cultured to an OD_600_ of 0.8, and before adding IPTG, selenomethionine was supplemented along with leucine, isoleucine, and valine to the final concentration of 50 µg/L, and lysine, threonine, and phenylalanine to 100 µg/L.

The trimer formation was checked by size exclusion chromatography on a Superdex-200 column (GE Healthcare) pre-equilibrated in a buffer with 20 mM Tris-HCl at pH 7.4, 50 mM NaCl and 5% glycerol.

### Crystallization and Data collection

The purified proteins were crystallized by sitting drop vapor diffusion method mixed 1:1 with reservoir solution. Crystals appeared in reservoir buffer containing 0.1 M Tris-HCl, pH 8.5, 50 mM magnesium acetate and 20% PEG4000 in 2 weeks at 18 °C. The crystals were frozen in a cryoprotectant consisting of the reservoir solution supplemented with 20% glycerol. Data were collected on the BL17U1 station of the Shanghai Synchrotron Radiation Facility (SSRF)^26^ and then were processed using the HKL2000, XDS and Xia2 software^27, 28^.

### Structure determination, Refinement and Analysis

One dataset for the selenomethionyl substituted MyRF DBD crystal was collected at the Se peak wavelength. The Se sites (4 sites, 4 Se per molecule) were identified using single anomalous diffraction from Se-Met by program Phenix.autosol. The model was built using Phenix.autobuild. The crystallographic refinement was performed by using all data to 2.46 Å resolution in COOT and PHENIX refinement programs^29, 30^. The orientations of the amino acid side chains and bound water molecules were modeled on the basis of 2F_obs__F_calc_ and F_obs__F_calc_ difference Fourier maps. Detailed data collection and refinement statistics are listed in Table 1. The model figures were generated with PyMol and CCP4mg. The interactions were analyzed with PyMol and LigPlus^31^.

**Table 1:** Data collection and refinement statistics.

MyRF DBD core	
**Data collection**	
Wavelength (Å)	0.9792
Space group	*P321*
Cell dimensions	
*a*, *b*, *c* (Å)	104.0 104.0 46.7
α, β, γ (°)	90.0 90.0 120.0
Resolution (Å)	34.75 – 2.46 (2.55 – 2.46)*
*R*_merge_^a^	0.097 (0.943)
CC1/2	0.998 (0.810)
*I*/σ(*I*)^b^	17.31 (2.44)
Completeness (%)	99.76 (98.31)
Redundancy	10.6 (10.1)
**Refinement**	
Resolution (Å)	34.03 – 2.46
Unique reflections	10,797 (1,044)
*R*_work_^c^/*R*_free_^d^	0.18/0.23 (0.34/0.42)
No. atoms	1,520
macromolecules	1,499
Water	21
B-factors	56.3
macromolecules	55.4
Water	46.5
R.m.s. deviations	
Bond lengths (Å)	0.010
Bond angles (°)	1.210
Ramachandran plot	
Favored regions (%)	97
Allowed refions (%)	3
Outliers (%)	0

*Values in parentheses are statistics for highest resolution shell.

^a^ *R*_*merge*_ *=* *Σ*_*hkl*_ *Σ*_*I*_ *|I*_*i*_ *(hkl)* − < *I*(*hkl*) > *| Σ*_*hkl*_ *Σ*_*i*_ *I*_*i*_ *(hkl)*, where *I*_*i*_ *(hkl)*_*i*_s the intensity of the *i*_th_ measurement of reflection *hkl*, i™ncluding symmetry-related reflections, and *<* *I(hkl)* *>* is their average.

^b^I /σ(I) = mean of intensity/σ(I) of unique reflections (after merging symmetry-related observations), σ(I) = standard deviation of reflection intensity I estimated from sample statistics.

^c^R_work_ = *Σ*_*h*_ *Σ*_*i*_ ||*F*_*o*_| − |*F*_*c*_||/*Σ* |*F*_*o*_|.

^d^R_free_ is R_work_ for ~10% of the reflection that were excluded from the refinement.

### The procedures used for generating the MyRF-DNA complex model

Superposition of MyRF DBD and Ndt80 (PDB code: 1mnn) was performed using UCSF Chimera program^32^ with the selection of best-aligning pair of chains between reference and match structure on MatchMaker. The DNA molecule in the Ndt80 structure was included in the superposition and modeled to the MyRF DBD. The trimeric MyRF-DNA complex was generated by symmetry operation of the single molecule.

## References

[1] Emery B (2009). Myelin gene regulatory factor is a critical transcriptional regulator required for CNS myelination. Cell.

[2] Compston A, Coles A (2008). Multiple sclerosis. Lancet.

[3] Aubourg P (1993). The leukodystrophies: a window to myelin. Nature genetics.

[4] McKenzie IA (2014). Motor skill learning requires active central myelination. Science.

[5] Stohr H, Marquardt A, White K, Weber BH (2000). cDNA cloning and genomic structure of a novel gene (C11orf9) localized to chromosome 11q12– > q13.1 which encodes a highly conserved, potential membrane-associated protein. Cytogenetics and cell genetics.

[6] Li Z, Park Y, Marcotte EM (2013). A Bacteriophage tailspike domain promotes self-cleavage of a human membrane-bound transcription factor, the myelin regulatory factor MYRF. PLoS biology.

[7] Bujalka H (2013). MYRF is a membrane-associated transcription factor that autoproteolytically cleaves to directly activate myelin genes. PLoS biology.

[8] Schulz EC (2010). Crystal structure of an intramolecular chaperone mediating triple-beta-helix folding. Nature structural & molecular biology.

[9] Senoo H, Araki T, Fukuzawa M, Williams JG (2013). A new kind of membrane-tethered eukaryotic transcription factor that shares an auto-proteolytic processing mechanism with bacteriophage tail-spike proteins. Journal of cell science.

[10] Lamoureux JS, Stuart D, Tsang R, Wu C, Glover JN (2002). Structure of the sporulation-specific transcription factor Ndt80 bound to DNA. The EMBO journal.

[11] Li H, Richardson WD (2015). Evolution of the CNS myelin gene regulatory program. Brain research.

[12] Hornig J (2013). The transcription factors Sox10 and Myrf define an essential regulatory network module in differentiating oligodendrocytes. PLoS genetics.

[13] Bork P, Holm L, Sander C (1994). The immunoglobulin fold. Structural classification, sequence patterns and common core. Journal of molecular biology.

[14] Holm L, Rosenstrom P (2010). Dali server: conservation mapping in 3D. Nucleic acids research.

[15] Petty TJ (2011). An induced fit mechanism regulates p53 DNA binding kinetics to confer sequence specificity. The EMBO journal.

[16] Huyen Y (2004). Structural differences in the DNA binding domains of human p53 and its C. elegans ortholog Cep-1. Structure.

[17] Becker S, Groner B, Muller CW (1998). Three-dimensional structure of the Stat3beta homodimer bound to DNA. Nature.

[18] Shrivastava T (2014). Structural basis of Ets1 activation by Runx1. Leukemia.

[19] Friedland N, Liou HL, Lobel P, Stock AM (2003). Structure of a cholesterol-binding protein deficient in Niemann-Pick type C2 disease. Proceedings of the National Academy of Sciences of the United States of America.

[20] Li X, Saha P, Li J, Blobel G, Pfeffer SR (2016). Clues to the mechanism of cholesterol transfer from the structure of NPC1 middle lumenal domain bound to NPC2. Proceedings of the National Academy of Sciences of the United States of America.

[21] Mann E, Mallette E, Clarke BR, Kimber MS, Whitfield C (2016). The Klebsiella pneumoniae O12 ATP-binding Cassette (ABC) Transporter Recognizes the Terminal Residue of Its O-antigen Polysaccharide Substrate. The Journal of biological chemistry.

[22] Ishikawa K (2015). Crystal structure of beta-galactosidase from Bacillus circulans ATCC 31382 (BgaD) and the construction of the thermophilic mutants. The FEBS journal.

[23] Senoo H, Wang HY, Araki T, Williams JG, Fukuzawa M (2012). An orthologue of the Myelin-gene Regulatory Transcription Factor regulates Dictyostelium prestalk differentiation. The International journal of developmental biology.

[24] Neudegger T, Verghese J, Hayer-Hartl M, Hartl FU, Bracher A (2016). Structure of human heat-shock transcription factor 1 in complex with DNA. Nature structural & molecular biology.

[25] Cherney LT, Cherney MM, Garen CR, James MN (2009). The structure of the arginine repressor from Mycobacterium tuberculosis bound with its DNA operator and Co-repressor, L-arginine. Journal of molecular biology.

[26] Wang QS (2015). The macromolecular crystallography beamline of SSRF. Nucl. Sci. Tech..

[27] Otwinowski, Z. & Minor, W. Processing of X-ray Diffraction Data Collected in Oscillation Mode. *Methods in enzymology* **276**, 307–326 C.W. Carter, Jr. & R. M. Sweet, Eds Academic Press (New York) (1997). 10.1016/S0076-6879(97)76066-X 27754618

[28] Kabsch WX (2010). Acta crystallographica. Section D, Biological crystallography.

[29] Adams, P. D. *et al*. PHENIX: a comprehensive Python-based system for macromolecular structure solution. *Acta crystallographica. Section D, Biological crystallography* **66**, 213-221, 10.1107/S0907444909052925 (2010). 10.1107/S0907444909052925 PMC2815670 20124702

[30] Emsley P, Lohkamp B, Scott WG, Cowtan K (2010). Features and development of Coot. Acta crystallographica. Section D, Biological crystallography.

[31] Wallace AC, Laskowski RA, Thornton JM (1995). LIGPLOT: a program to generate schematic diagrams of protein-ligand interactions. Protein engineering.

[32] Pettersen EF (2004). UCSF Chimera–a visualization system for exploratory research and analysis. Journal of computational chemistry.

